# Dataset describing the influence of culture conditions on the bioreduction of organic acids to alcohols by *Thermoanaerobacter pseudethanolicus*

**DOI:** 10.1016/j.dib.2023.109962

**Published:** 2023-12-15

**Authors:** Johann Orlygsson, Sean Michael Scully

**Affiliations:** Faculty of Natural Resource Science, University of Akureyri, Borgir v. Nordurslod, 600 Akureyri, Iceland

**Keywords:** Thermophilic bacteria, Fermentation, Reduction, Anaerobic

## Abstract

The dataset describes the influence of culture conditions on the bioreduction of organic acids by *Thermoanaerobacter pseudethanolicus* as reported in [1]. The data shows that during glucose fermentation of *Thermoanaerobacter pseudethanolicus* the reducing equivalents are not only converted to ethanol and hydrogen but also, in the presence of carboxylic acids (C2–C6), to its corresponding alcohol. To maximize the alcohol production produced from their carboxylic acid, several experiments were performed to investigate the effect of various environmental factors (initial glucose concentration, pH, liquid–gas phase ratio, and inhibitory effects of alcohols) on growth. A kinetic experiment of glucose in the absence and presence of selected fatty acids are also presented as are data on selected enzyme activities related to alcohols and aldehydes and a time course study of the reduction of ^13^C1 labeled butyrate using glucose as a carbon source.

Specifications Table

**Table:** 

Subject	Biology
Specific subject area	Microbiology
Data format	Raw
Type of data	Table, figure
Data collection	The bacteria investigated was cultivated under various environmental conditions and both substrate and end-product formation analysed using GC-FID, Perkin Elmer Clarus 580, GC-TCD, Perkin Elmer Autosystem XL, UV–visible Spectroscopy, Bioscreen C (GrowthCurves Ltd, Finland) and Shimadzu UV-1800 UV–visible Spectrometer, Bruker AV400 NMR Spectrometer.
Data source location	Institution: University of Akureyri Region: Akureyri, Iceland
Data accessibility	Repository name: Mendeley Data identification number: 10.17632/wxrd9fh9xt.1 Direct URL to data: https://data.mendeley.com/datasets/wxrd9fh9xt/1
Related research article	S.M. Scully, A.E. Brown, Y. Mueller-Hilger, A.B. Ross, J. Örlygsson, Influence of Culture Conditions on the Bioreduction of Organic Acids to Alcohols by *Thermoanaerobacter pseudoethanolicus*, Microorganisms. 9 (2021) 1–24. https://doi.org/10.3390/microorganisms9010162

## 1 Value of the Data


 

• The data presents end products from the fermentation glucose in the presence of volatile fatty acids (formate, acetate, 1-propionate, 1-butyrate, 2-methyl-1-propionate, 1-pentanoate, 3-methyl-1-butyrate, 2-methyl-1-butyrate, 1-hexanoate) by *Thermoanaerobacter pseudethanolicus.*
• The data set shows the influence of culture parameters on the fermentation of glucose in the presence of volatile fatty and under different environmental conditions. A kinetic experiment showing the formation of 1-butanol from 1-butyrate and 3-methyl-1-butanol from 3-methyl-1-butyrate.
• Could be useful for producing longer chain alcohols from low-value volatile fatty acids found in waste materials.

## 2 Data Description

*Thermoanaerobacter pseudethanolicus* ferments glucose predominantly to ethanol but also to minor amounts of acetate and hydrogen [1]. Growth on glucose in the presence of exogenously added volatile fatty acids (20 mM) leads to less amounts of ethanol and an increase in acetate formation and the added fatty acid is converted to their corresponding alcohol [1]. The influence of various environmental parameters are known to result in a change in end-product formation, such as using different liquid–gas phase ratios and pH was investigated in batch culture in the present study.

**Fig. 1:**
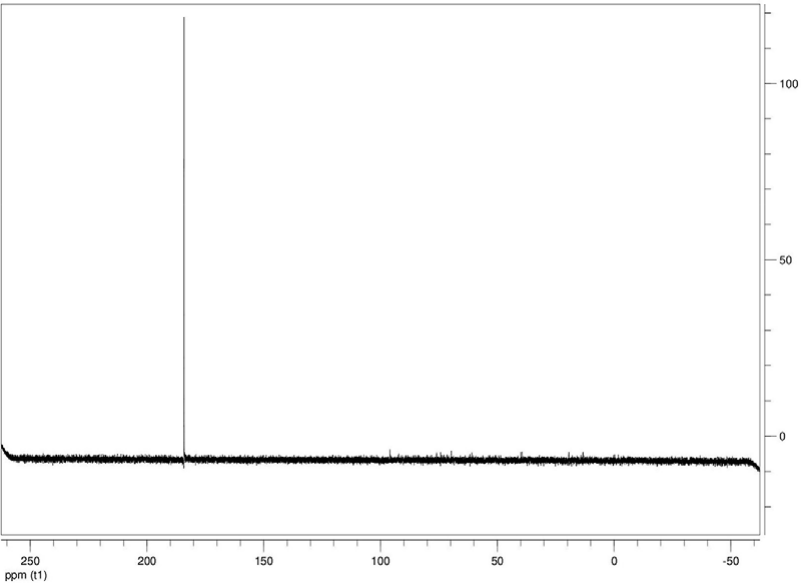
Spectrogram of ^13^C NMR spectra from *T. pseudethanolicus* culture broth containing 20 mM of ^13^C1 butyrate and 20 mM of glucose at the start of the fermentation (0 h). Peak at 183.3 ppm can be attributed to the C1 position of butyrate.

**Fig. 2:**
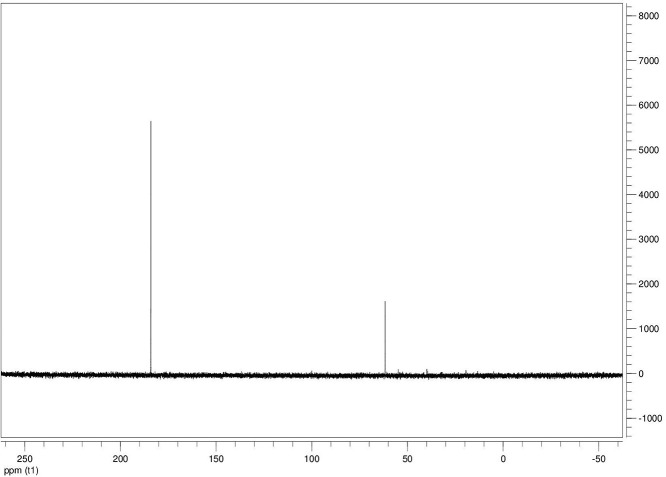
Spectrogram of ^13^C NMR spectra from *T. pseudethanolicus* culture broth containing 20 mM of ^13^C1 butyrate and 20 mM of glucose after 4 h of fermentation. Peak at 183.3 ppm can be attributed to the C1 position of butyrate and the peak at 60.0 ppm is the C1 position of 1-butanol.

**Fig. 3:**
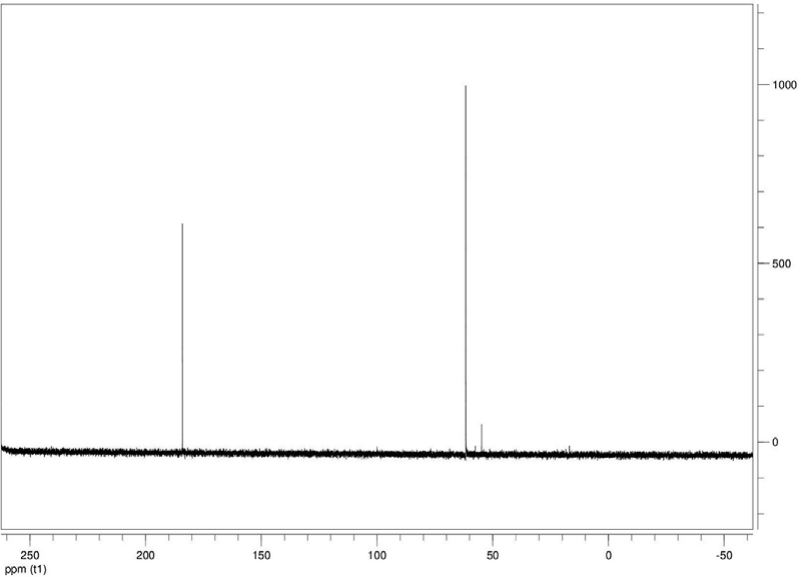
Spectrogram of ^13^C NMR spectra from *T. pseudethanolicus* culture broth containing 20 mM of ^13^C1 butyrate and 20 mM of glucose after 8 h of fermentation. Peak at 183.3 ppm can be attributed to the C1 position of butyrate and the peak at 60.0 ppm is the C1 position of 1-butanol.

**Fig. 4:**
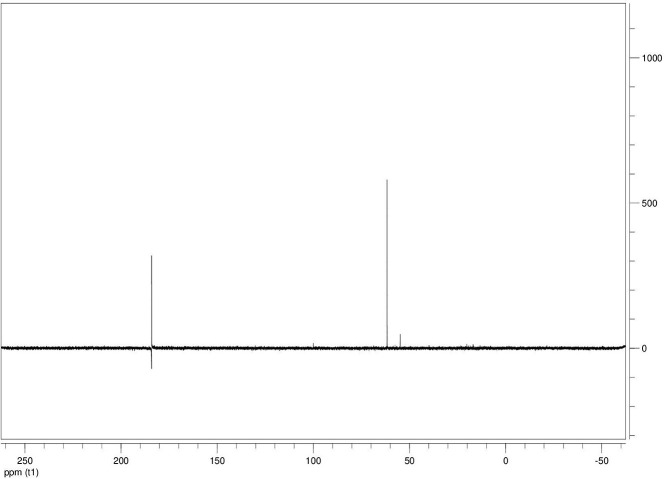
Spectrogram of ^13^C NMR spectra from *T. pseudethanolicus* culture broth containing 20 mM of ^13^C1 butyrate and 20 mM of glucose after 12 h of fermentation. Peak at 183.3 ppm can be attributed to the C1 position of butyrate and the peak at 60.0 ppm is the C1 position of 1-butanol.

**Fig. 5:**
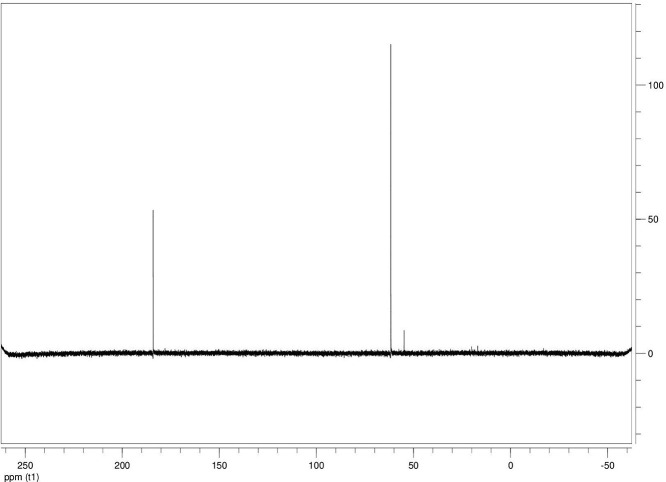
Spectrogram of ^13^C NMR spectra from *T. pseudethanolicus* culture broth containing 20 mM of ^13^C1 butyrate and 20 mM of glucose after 16 h of fermentation. Peak at 183.3 ppm can be attributed to the C1 position of butyrate and the peak at 60.0 ppm is the C1 position of 1-butanol.

**Fig. 6:**
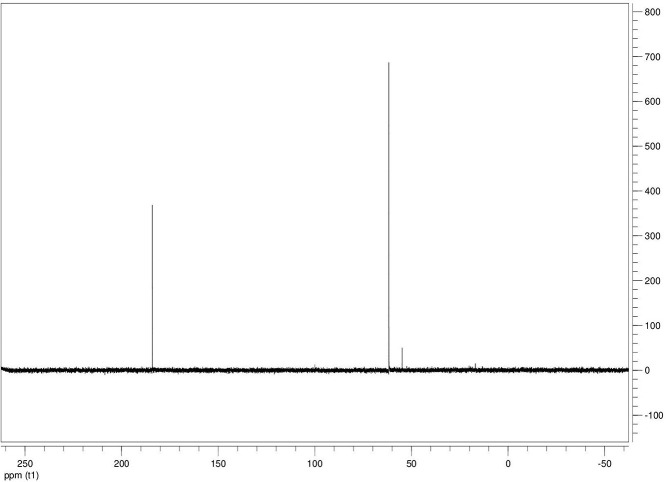
Spectrogram of ^13^C NMR spectra from *T. pseudethanolicus* culture broth containing 20 mM of ^13^C1 butyrate and 20 mM of glucose after 24 h of fermentation. Peak at 183.3 ppm can be attributed to the C1 position of butyrate and the peak at 60.0 ppm is the C1 position of 1-butanol.

**Fig. 7:**
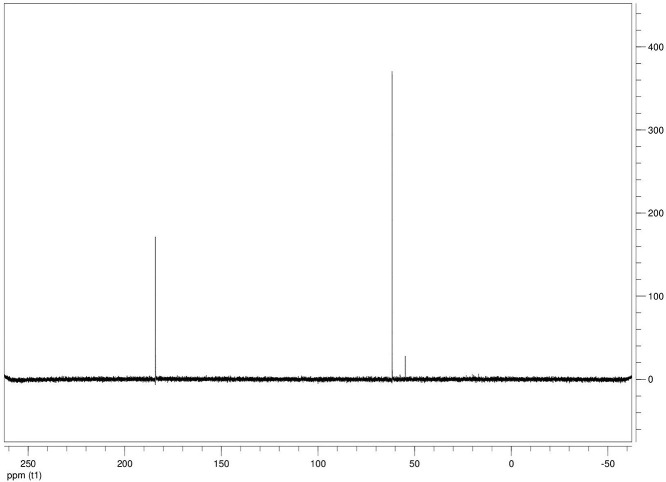
Spectrogram of ^13^C NMR spectra from *T. pseudethanolicus* culture broth containing 20 mM of ^13^C1 butyrate and 20 mM of glucose after 30 h of fermentation. Peak at 183.3 ppm can be attributed to the C1 position of butyrate and the peak at 60.0 ppm is the C1 position of 1-butanol.

**Fig. 8:**
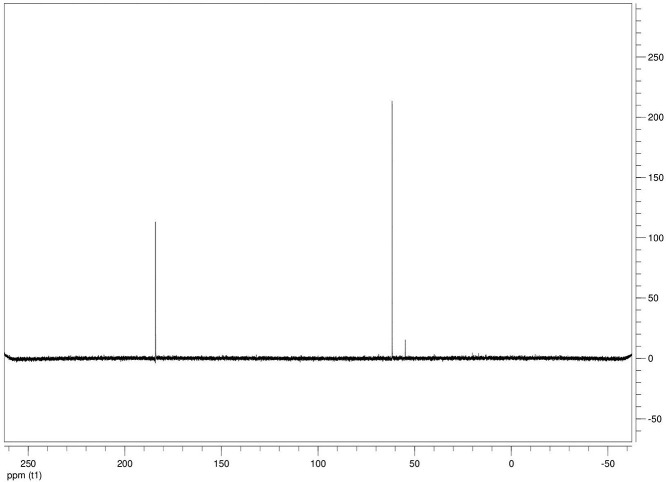
Spectrogram of ^13^C NMR spectra from *T. pseudethanolicus* culture broth containing 20 mM of ^13^C1 butyrate and 20 mM of glucose after 36 h of fermentation. Peak at 183.3 ppm can be attributed to the C1 position of butyrate and the peak at 60.0 ppm is the C1 position of 1-butanol.

**Fig. 9:**
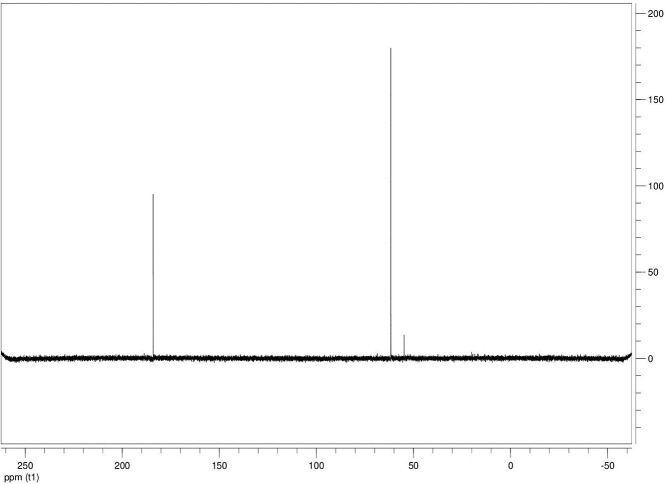
Spectrogram of ^13^C NMR spectra from *T. pseudethanolicus* culture broth containing 20 mM of ^13^C1 butyrate and 20 mM of glucose after 48 h of fermentation. Peak at 183.3 ppm can be attributed to the C1 position of butyrate and the peak at 60.0 ppm is the C1 position of 1-butanol.

**Fig. 10:**
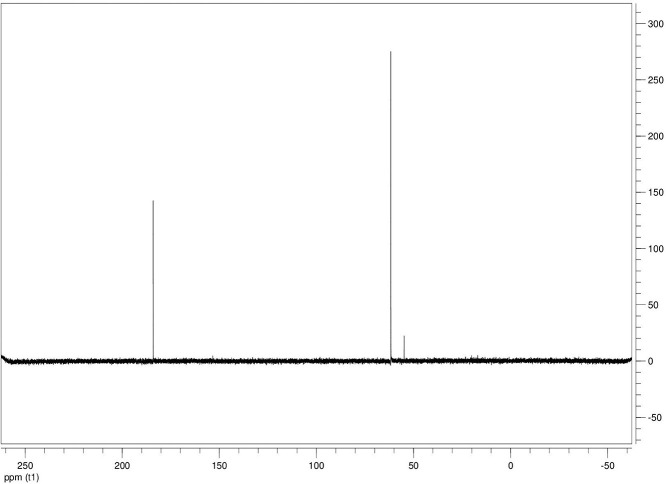
Spectrogram of ^13^C NMR spectra from *T. pseudethanolicus* culture broth containing 20 mM of ^13^C1 butyrate and 20 mM of glucose after 60 h of fermentation. Peak at 183.3 ppm can be attributed to the C1 position of butyrate and the peak at 60.0 ppm is the C1 position of 1-butanol.

**Fig. 11:**
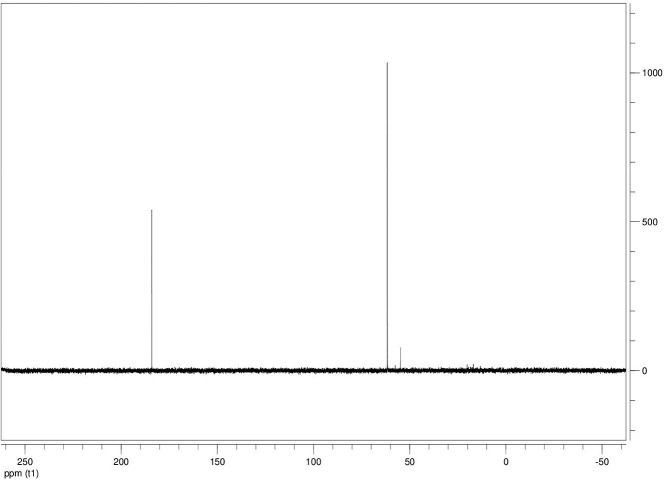
Spectrogram of ^13^C NMR spectra from *T. pseudethanolicus* culture broth containing 20 mM of ^13^C1 butyrate and 20 mM of glucose after 72 h of fermentation. Peak at 183.3 ppm can be attributed to the C1 position of butyrate and the peak at 60.0 ppm is the C1 position of 1-butanol.

The dataset contains seven tables (Tables final.docx), six of which detail fermentation data, namely metabolic end products such as alcohols, fatty acids, and hydrogen, while the remaining table contains enzyme activities towards selected alcohols and aldehyde substrates. Each line details the experimental conditions for a given experiment with a data point for the analyte concentration in mmol per L presented as the average±standard deviation measured at the indicated time. Additionally, one table summarizes the enzymatic activity of crude cell lysates towards alcohol and aldehyde substrates using NAD^+^ or NADP^+^ as a cofactor. Table 1 details the conversion rate of the fatty acid conversion to alcohols using C1–C6 carbon fatty acids. Table 2 displays kinetic conversion of glucose alone and in the presence of 1-butyrate and 3-methyl-1-butyrate. Table 3 shows the conversion of selected fatty acids to their corresponding alcohols in the presence of glucose at different initial pH values. The effect of using different L-G ratio on the same fatty acids are shown in Table 4. Table 5 shows the effect of increasing glucose concentrations on the conversion of 1-propionate, 1-butyrate, and 2-methyl-1-propionate to their corresponding alcohols. Table 6 shows the inhibitory effects of various compounds added in different concentrations to an active culture of *T. pseudethanolicus*. The volumetric activities of oxidative enzyme reactions using NAD^+^ and NADP^+^ as a cofactor when *T. pseudethanolicus* is cultivated on either glucose (20 mM) or glucose supplemented with selected carboxylic acids are presented in Table 7. Fig. 1, Fig. 2, Fig. 3, Fig. 4, Fig. 5, Fig. 6, Fig. 7, Fig. 8, Fig. 9, Fig. 10, Fig. 11 through 11 detail the ^13^C NMR spectra of *T. pseudethanolicus* cultivated on glucose (20 mM) supplemented with ^13^C1-labled butyrate over a period of 72 h.

**Table 1:** End product formation after 5 days of cultivation from cultures of *T. pseudethanolicus* containing glucose (20 mM) and of exogenously added carboxylic acid (RCOOH; 20 mM) and its conversion to its corresponding short-chain alcohol (ROH). Values represent the average of triplicate fermentations with standard deviation.

	**Analyte** (mmol/L)								
Substrate (20 mM) + carboxylic acid (20 mM)	Hydrogen	Ethanol	Alcohol	Acetate	Carboxylic acid	Carboxylic acid conversion (%)	ROH/RCOOH Ratio	Optical Density (600 nm)	Carbon balance (%)
Control (yeast extract)	0.18 ± 0.02	1.38 ± 0.18	ND	2.92 ± 0.17	ND	ND	ND	0.06 ± 0.02	ND
Glucose + Formate	2.50 ± 0.15	23.22 ± 4.64	ND	5.10 ± 1.84	ND	ND	ND	0.33 ± 0.02	70.8
Glucose + Acetate	1.45 ± 0.04	25.70 ± 3.28	ND	34.78 ± 3.27	ND	ND	ND	0.35 ± 0.04	101.2
Glucose + 1-Propionate	1.58 ± 0.33	16.49 ± 2.03	6.62 ± 0.58	13.01 ± 0.58	10.55 ± 0.38	47.2	0,63	0.35 ± 0.01	73.8
Glucose + 1-Butyrate	1.73 ± 1.02	22.90 ± 3.00	9.14 ± 1.17	13.01 ± 0.43	6.38 ± 0.28	68.1	1,43	0.30 ± 0.09	89.7
Glucose + 2-Methyl-1-Propionate	1.27 ± 0.29	20.36 ± 1.10	9.94 ± 0.83	22.34 ± 2.55	10.24 ± 1.17	48.6	0,97	0.31 ± 0.04	106.7
Glucose + 1-Pentanoate	1.42 ± 1.13	19.74 ± 1.77	11.58 ± 0.75	16.91 ± 1.68	8.41 ± 0.50	58.0	1.37	0.35 ± 0.01	91.6
Glucose + 3-Methyl-1-Butyrate	2.76 ± 0.26	21.42 ± 4.56	4.18 ± 0.90	9.36 ± 0.34	11.14 ± 1.63	44.3	0.38	0.38 ± 0.04	76.9
Glucose + 2-Methyl-1-Butyrate	2.15 ± 0.06	18.87 ± 1.00	7.22 ± 0.25	19.86 ± 1.03	12.50 ± 0.50	37.5	0.58	0.38 ± 0.01	96.8
Glucose + 1-Hexanoate	1.15 ± 0.31	31.73 ± 5.70	6.69 ± 1.96	12.00 ± 2.06	12.23 ± 2.99	38.9	0.55	0.32 ± 0.09	109.2

**Table 2:** Time-course studies of fermentation of 20 mM glucose, 20 mM 1-butyrate + 20 mM glucose, and 20 mM 3-methyl-1-butyrate + 20 mM glucose by *T. pseudethanolicus*. Values represent the average of triplicate fermentations with standard deviation presented as error bars.

		**Analyte** (mmol/L)									
Substrate	Time (h)	Hydrogen	Ethanol	Alcohol	Acetate	Carboxylic acid	Glucose (remaining)	Carboxylic acid conversion (%)	Glucose consumed (%)	Optical Density (600 nm)	Carbon balance (%)
Yeast extract (control)	0	0.00 ± 0.00	0.00 ± 0.00	ND	0.00 ± 0.00	ND	ND	NA	NA	0.00 ± 0.00	ND
	4	0.24 ± 0.07	0.12 ± 0.20	ND	0.45 ± 0.07	ND	ND	NA	NA	0.06 ± 0.01	ND
	8	0.48 ± 0.13	0.38 ± 0.03	ND	1.34 ± 0.03	ND	ND	NA	NA	0.10 ± 0.05	ND
	12	0.75 ± 0.20	0.57 ± 0.13	ND	2.21 ± 0.25	ND	ND	NA	NA	0.11 ± 0.01	ND
	18	1.41 ± 0.29	0.91 ± 0.18	ND	2.51 ± 0.18	ND	ND	NA	NA	0.12 ± 0.02	ND
	24	2.84 ± 0.25	1.13 ± 0.34	ND	2.92 ± 0.01	ND	ND	NA	NA	0.10 ± 0.06	ND
	30	2.34 ± 0.37	1.38 ± 0.39	ND	2.99 ± 0.17	ND	ND	NA	NA	0.10 ± 0.00	ND
	36,5	2.22 ± 0.48	1.51 ± 0.30	ND	3.09 ± 0.24	ND	ND	NA	NA	0.07 ± 0.01	ND
	48	2.47 ± 0.31	1.48 ± 0.67	ND	3.17 ± 0.19	ND	ND	NA	NA	0.08 ± 0.01	ND
	120	2.43 ± 0.15	1.28 ± 0.24	ND	3.04 ± 0.16	ND	ND	NA	NA	0.07 ± 0.04	ND

Glucose (20 mM)	0	0.00 ± 0.00	0.00 ± 0.00	ND	0.00 ± 0.00	ND	20.00 ± 0.00	NA	0.0	0.00 ± 0.00	100.0
	4	0.00 ± 0.00	1.06 ± 0.31	ND	1.50 ± 0.20	ND	20.00 ± 0.00	NA	0.0	0.05 ± 0.00	103.8
	8	0.22 ± 0.02	1.52 ± 0.52	ND	2.68 ± 0.90	ND	18.30 ± 1.10	NA	8.5	0.09 ± 0.01	102.0
	12	0.74 ± 0.20	8.88 ± 1.65	ND	6.66 ± 1.06	ND	14.70 ± 1.47	NA	26.5	0.22 ± 0.02	112.4
	18	1.24 ± 0.20	13.51 ± 0.52	ND	7.21 ± 1.34	ND	9.10 ± 1.53	NA	54.5	0.34 ± 0.02	97.4
	24	1.55 ± 0.38	29.55 ± 0.49	ND	7.68 ± 0.62	ND	2.40 ± 0.48	NA	88.0	0.31 ± 0.06	105.1
	30	2.08 ± 0.42	31.30 ± 3.52	ND	5.73 ± 1.28	ND	2.30 ± 0.57	NA	88.5	0.30 ± 0.02	104.1
	36,5	1.76 ± 0.36	29.80 ± 0.58	ND	5.71 ± 1.55	ND	0.70 ± 0.18	NA	96.5	0.30 ± 0.01	92.3
	48	2.62 ± 0.47	35.04 ± 2.13	ND	6.88 ± 1.74	ND	0.00 ± 0.00	NA	100	0.25 ± 0.01	104.7
	120	2.46 ± 0.18	30.22 ± 0.54	ND	6.490.67	ND	0.00 ± 0.00	NA	100	0.12 ± 0.05	91.8
											
Glucose (20 mM)	0	0.00 ± 0.00	0.00 ± 0.00	0.00 ± 0.00 (1-BuOH)	0.00 ± 0.00	20.00 ± 0.00	20.00 ± 0.00	0.0	0.0	0.00 ± 0.00	100.0
+ 1-butryate (20 mM)	4	0.00 ± 0.00	0.36 ± 0.24	0.00 ± 0.00 (1-BuOH)	1.00 ± 0.28	19.68 ± 0.25	20.00 ± 0.00	0.0	0.0	0.06 ± 0.01	101.7
	8	0.17 ± 0.12	1.45 ± 0.15	0.40 ± 0.08 (1-BuOH)	2.50 ± 0.16	18.61 ± 1.15	17.98 ± 0.53	2.0	10.1	0.11 ± 0.02	98.2
	12	0.64 ± 0.05	12.50 ± 1.66	3.47 ± 0.72 (1-BuOH)	6.36 ± 0.99	14.45 ± 2.25	13.56 ± 0.91	17.4	32.2	0.37 ± 0.06	106.5
	18	0.82 ± 0.17	18.15 ± 1.24	4.23 ± 0.23 (1-BuOH)	9.47 ± 0.37	12.34 ± 1.01	9.54 ± 1.22	21.2	52.3	0.42 ± 0.02	105.5
	24	1.11 ± 0.10	21.88 ± 4.80	8.73 ± 2.03 (1-BuOH)	13.85 ± 0.74	8.69 ± 1.96	3.21 ± 0.32	43.7	84.0	0.25 ± 0.01	99.3
	30	1.13 ± 0.30	24.82 ± 1.78	10.11 ± 0.72 (1-BuOH)	14.89 ± 0.66	9.79 ± 1.33	2.47 ± 0.19	50.6	87.7	0.17 ± 0.01	107.6
	36,5	1.29 ± 0.08	27.27 ± 0.30	10.68 ± 1.41 (1-BuOH)	16.15 ± 2.87	9.83 ± 1.80	0.60 ± 0.23	53.4	97.0	0.19 ± 0.05	108.6
	48	1.44 ± 0.09	27.34 ± 3.65	10.43 ± 1.53 (1-BuOH)	15.16 ± 1.37	6.50 ± 1.60	0.00 ± 0.00	52.2	100.0	0.15 ± 0.00	99.1
	120	1.25 ± 0.41	26.22 ± 1.18	11.19 ± 0.23 (1-BuOH)	15.87 ± 0.93	6.43 ± 1.10	0.00 ± 0.00	56.0	100.0	0.13 ± 0.01	99.5
											
Glu (20 mM) +	0	0.00 ± 0.00	0.00 ± 0.00	0.00 ± 0.00 (3-Me-1-BuOH)	0.00 ± 0.00	20.00 ± 0.00	20.00 ± 0.00	0.0	0.0	0.00 ± 0.00	100.0
3-me-1-butryate (20 mM)	4	0.00 ± 0.00	0.46 ± 0.07	0.00 ± 0.00 (3-Me-1-BuOH)	1.29 ± 0.13	20.42 ± 1.05	20.00 ± 0.00	0.0	0.0	0.05 ± 0.00	103.6
	8	0.32 ± 0.04	1.64 ± 0.37	0.00 ± 0.00 (3-Me-1-BuOH)	2.49 ± 0.11	19.68 ± 2.33	18.30 ± 1.10	0.0	8.5	0.09 ± 0.00	100.7
	12	0.43 ± 0.04	8.94 ± 1.72	0.00 ± 0.00 (3-Me-1-BuOH)	4.94 ± 0.64	18.52 ± 2.88	14.70 ± 1.47	0.0	26.5	0.35 ± 0.04	103.0
	18	0.84 ± 0.21	15.13 ± 2.16	1.13 ± 0.07 (3-Me-1-BuOH)	8.52 ± 0.54	14.22 ± 1.34	9.10 ± 1.53	5.7	54.5	0.38 ± 0.03	95.3
	24	1.73 ± 0.16	26.55 ± 0.87	2.26 ± 0.14 (3-Me-1-BuOH)	12.22 ± 1.10	12.99 ± 1.25	2.40 ± 0.48	11.3	88.0	0.43 ± 0.03	98.0
	30	1.87 ± 0.16	26.93 ± 2.15	2.24 ± 0.13 (3-Me-1-BuOH)	12.72 ± 0.41	13.18 ± 1.65	2.30 ± 0.57	11.3	88.5	0.36 ± 0.04	102.6
	36,5	1.59 ± 0.17	31.13 ± 0.93	2.29 ± 0.10 (3-Me-1-BuOH)	13.26 ± 0.64	13.66 ± 1.18	0.70 ± 0.18	11.5	96.5	0.26 ± 0.08	102.9
	48	2.15 ± 0.16	27.66 ± 1.65	2.31 ± 0.10 (3-Me-1-BuOH)	13.05 ± 0.14	13.82 ± 1.36	0.00 ± 0.00	11.6	100.0	0.24 ± 0.03	94.7
	120	1.64 ± 0.40	27.87 ± 0.72	6.16 ± 0.27 (3-Me-1-BuOH)	13.44 ± 0.80	13.12 ± 0.72	0.00 ± 0.00	30.8	100.0	0.20 ± 0.00	101.0

ND – Not detected; NA – Not applicable.

**Table 3:** Impact of initial pH on end product formation and carboxylic acid conversion by *T. pseudethanolicus* after 5 days.

		**Analyte** (mmol/L)									
Substrate (20 mM) + carboxylic acid (20 mM)	Initial pH	Hydrogen	Ethanol	Alcohol	Acetate	Carboxylic acid	Glucose	Carboxylic acid conversion (%)	ROH/RCOOH Ratio	Optical Density (600 nm)	Carbon balance (%)
Glucose +1-propionate	5.0	2.01 ± 0.14	11.17 ± 0.38	5.81±0.31 (1-PrOH)	13.21 ± 0.67	13.78 ± 1.03	5.85 ± 0.51	29.1	0.42	0.17 ± 0.02	92.8
	5.5	1.53 ± 0.37	14.43 ± 1.01	6.54 ± 0.67 (1-PrOH)	14.14 ± 0.54	12.81 ± 0.48	2.37 ± 0.27	32.7	0.51	0.24 ± 0.08	87.8
	6.0	1.27 ± 0.07	18.14 ± 1.17	8.21 ± 1.01 (1-PrOH)	10.64 ± 1.01	11.17 ± 0.60	0.94 ± 0.07	41.1	0.74	0.29 ± 0.20	83.4
	6.5	1.23 ± 0.15	22.37 ± 0.39	10.13 ± 0.42 (1-PrOH)	13.23 ± 0.48	13.21 ± 0.77	0.00 ± 0.00	50.7	0.77	0.25 ± 0.11	98.2
	7.0	1.17 ± 0.15	21.10 ± 0.85	8.24 ± 0.28 (1-PrOH)	14.54 ± 0.39	12.77 ± 0.50	0.00 ± 0.00	41.2	0.65	0.36 ± 0.03	94.4
	7.5	1.37 ± 0.42	23.37 ± 0.68	8.34 ± 0.39 (1-PrOH)	12.34 ± 0.14	11.31 ± 0.76	0.00 ± 0.00	41.7	0.74	0.34 ± 0.03	92.3
	8.0	0.81 ± 0.29	20.17 ± 0.72	7.28 ± 0.22 (1-PrOH)	13.31 ± 0.32	13.58 ± 0.36	0.71 ± 0.09	36.4	0.54	0.32 ± 0.04	92.9
	8.5	0.38 ± 0.21	19.41 ± 0.43	6.87 ± 0.26 (1-PrOH)	7.81 ± 0.37	13.36 ± 0.57	1.89 ± 0.2	34.4	0.51	0.28 ± 0.02	85.4
Glucose +1-butyrate	5.0	1.43 ± 0.07	10.00 ± 0.58	6.05 ± 0.42 (1-BuOH)	11.20 ± 0.36	13.20 ± 1.03	5.91 ± 1.13	28.1	0.46	0.17 ± 0.02	87.1
	5.5	1.07 ± 0.23	13.80 ± 1.27	6.91 ± 0.83 (1-BuOH)	13.37 ± 0.28	13.72 ± 0.48	2.02 ± 0.39	31.4	0.50	0.24 ± 0.08	86.4
	6.0	1.22 ± 0.12	21.17 ± 1.50	7.74 ± 1.63 (1-BuOH)	9.57 ± 1.36	7.62 ± 0.60	1.12 ± 0.18	38.7	1.01	0.29 ± 0.05	80.6
	6.5	1.21 ± 0.05	22.42 ± 1.64	10.00 ± 0.45 (1-BuOH)	11.82 ± 0.22	7.42 ± 0.77	0.34 ± 0.07	50.0	1.35	0.25 ± 0.11	87.2
	7.0	1.18 ± 0.03	19.50 ± 1.53	8.91 ± 0.31 (1-BuOH)	11.08 ± 2.14	7.77 ± 0.50	0.12 ± 0.04	44.6	1.15	0.36 ± 0.03	79.2
	7.5	1.39 ± 0.11	21.72 ± 1.40	9.11 ± 0.36 (1-BuOH)	10.52 ± 0.13	7.89 ± 0.76	0.05 ± 0.01	45.5	1.15	0.34 ± 0.03	82.2
	8.0	0.84 ± 0.19	18.43 ± 0.69	8.26 ± 0.25 (1-BuOH)	12.31 ± 0.43	11.61 ± 0.36	1.81 ± 0.25	41.3	0.71	0.32 ± 0.04	90.4
	8.5	0.44 ± 0.05	19.24 ± 1.24	7.03 ± 0.17 (1-BuOH)	8.31 ± 0.62	12.41 ± 0.57	2.01 ± 0.23	35.2	0.57	0.28 ± 0.02	85.0
Glucose +2-methyl-1-butyrate	5.0	1.01 ± 0.15	15.21 ± 0.89	2.89 ± 0.52 (2-Me-1-BuOH)	8.13 ± 0.37	15.39 ± 0.65	5.31 ± 0.14	14.5	0.19	0.18 ± 0.05	87.1
	5.5	1.21 ± 0.09	19.31 ± 0.17	3.81 ± 0.33 (2-Me-1-BuOH)	9.11 ± 0.48	14.31 ± 0.81	3.45 ± 0.14	19.1	0.27	0.22 ± 0.04	89.1
	6.0	0.88 ± 0.18	28.76 ± 1.43	4.33 ± 0.19 (2-Me-1-BuOH)	8.41 ± 0.54	12.90 ± 1.46	0.00 ± 0.00	24.1	0.49	0.28 ± 0.06	90.7
	6.5	0.95 ± 0.13	28.96 ± 0.10	4.42 ± 0.20 (2-Me-1-BuOH)	8.06 ± 1.49	12.91 ± 2.34	0.00 ± 0.00	22.1	0.22	0.30 ± 0.04	90.6
	7.0	0.96 ± 0.13	27.52 ± 0.75	4.18 ± 0.37 (2-Me-1-BuOH)	8.22 ± 0.47	11.22 ± 0.65	0.10 ± 0.00	20.9	0.37	0.33 ± 0.03	85.6
	7.5	1.08 ± 0.07	23.41 ± 0.96	4.52 ± 0.34 (2-Me-1-BuOH)	9.16 ± 0.45	10.55 ± 2.02	0.31 ± 0.05	22.6	0.43	0.53 ± 0.18	80.4
	8.0	0.92 ± 0.21	22.78 ± 1.23	3.17 ± 0.28 (2-Me-1-BuOH)	9.01 ± 0.38	15.81 ± 0.92	1.24 ± 0.31	15.9	0.20	0.46 ± 0.10	88.8
	8.5	0.14 ± 0.02	24.32 ± 0.69	2.94 ± 0.72 (2-Me-1-BuOH)	6.84 ± 0.31	16.27 ± 0.61	3.21 ± 0.47	14.7	0.18	0.31 ± 0.04	94.7
Glucose +3-methyl-1-butyrate	5.0	0.71 ± 0.03	15.21 ± 1.12	2.71 ± 0.38 (3-Me-1-BuOH)	8.13 ± 0.37	17.51 ± 0.27	4.89 ± 0.61	13.6	0.15	0.15 ± 0.04	88.9
	5.5	1.09 ± 0.06	19.31 ± 1.30	3.34 ± 0.47 (3-Me-1-BuOH)	9.11 ± 0.48	16.27 ± 0.35	3.22 ± 0.27	16.7	0.21	0.21 ± 0.02	90.8
	6.0	0.82 ± 0.03	28.76 ± 1.43	4.21 ± 0.24 (3-Me-1-BuOH)	8.41 ± 0.54	16.14 ± 0.80	0.23 ± 0.04	21.1	0.26	0.28 ± 0.06	96.6
	6.5	0.99 ± 0.07	28.96 ± 0.10	5.01 ± 0.31 (3-Me-1-BuOH)	8.06 ± 1.49	15.67 ± 2.34	0.00 ± 0.00	25.1	0.32	0.30 ± 0.04	96.2
	7.0	1.07 ± 0.07	27.52 ± 0.75	4.15 ± 0.24 (3-Me-1-BuOH)	8.22 ± 0.47	11.22 ± 0.65	0.00 ± 0.00	20.8	0.37	0.33 ± 0.03	85.2
	7.5	1.21 ± 0.05	20.97 ± 0.96	4.34 ± 0.34 (3-Me-1-BuOH)	9.16 ± 0.45	16.54 ± 1.07	0.00 ± 0.00	21.7	0.26	0.53 ± 0.18	85.0
	8.0	0.81 ± 0.14	24.14 ± 1.23	2.64 ± 0.23 (3-Me-1-BuOH)	9.01 ± 0.38	17.56 ± 0.27	0.89 ± 0.03	13.2	0.15	0.48 ± 0.07	91.9
	8.5	0.15 ± 0.03	24.32 ± 1.47	2.40 ± 0.51 (3-Me-1-BuOH)	6.84 ± 0.31	17.27 ± 0.30	2.87 ± 0.17	12.0	0.14	0.32 ± 0.04	94.3

**Table 4:** Impact of liquid-gas phase ratios on end product formation after 5 days from cultures of *T. pseudethanolicus*. Values represent the average of triplicates  ±  standard deviation.

		**Analyte** (mmol/L)								
Substrate (20 mM) + carboxylic acid (20 mM)	L-G ratio	Hydrogen	Ethanol	Alcohol	Acetate	Carboxylic acid	Carboxylic acid conversion (%)	ROH/RCOOH Ratio	Optical Density (600 nm)	Carbon balance (%)
Glucose	0.09	0.96 ± 0.19	25.84 ± 3.26	ND	14.65 ± 3.81	NA	ND	ND	0.26 ± 0.02	101.2
	0.34	2.04 ± 0.35	25.25 ± 1.14	ND	7.19 ± 0.32	NA	ND	ND	0.28 ± 0.01	81.1
	1.00	3.72 ± 0.50	30.43 ± 2.26	ND	6.94 ± 2.25	NA	ND	ND	0.24 ± 0.02	93.4
	2.12	6.03 ± 0.27	27.39 ± 2.26	ND	4.21 ± 0.09	NA	ND	ND	0.22 ± 0.00	79.0
	5.26	4.65 ± 0.42	32.31 ± 3.46	ND	3.62 ± 0.40	NA	ND	ND	0.22 ± 0.01	89.8
										
Glucose +1-propionate	0.09	1.05 ± 0.15	13.52 ± 0.98	11.18 ± 0.64 (1-PrOH)	22.43 ± 1.04	10.45 ± 0.82	55.9	1.07	0.18 ± 0.02	96.0
	0.34	0.33 ± 0.27	9.10 ± 1.73	8.96 ± 1.06 (1-PrOH)	15.07 ± 0.57	12.24 ± 0.03	44.8	0.73	0.06 ± 0.01	84.9
	1.00	0.75 ± 0.11	10.06 ± 0.30	9.57 ± 0.34 (1-PrOH)	14.62 ± 0.50	12.00 ± 0.19	47.9	0.80	0.07 ± 0.02	77.1
	2.12	1.12 ± 0.09	11.17 ± 0.23	10.38 ± 0.30 (1-PrOH)	15.34 ± 0.34	11.58 ± 0.39	51.9	0.90	0.08 ± 0.02	92.2
	5.26	1.95 ± 0.19	10.96 ± 0.39	10.48 ± 0.25 (1-PrOH)	16.23 ± 1.08	12.49 ± 0.23	52.4	0.84	0.12 ± 0.05	94.2
										
Glucose +1-butyrate	0.09	0.59 ± 0.08	23.28 ± 0.36	11.22 ± 0.29 (1-BuOH)	19.40 ± 0.68	13.45 ± 1.18	56.1	0.83	0.25 ± 0.04	89.8
	0.34	0.96 ± 0.05	24.71 ± 0.97	11.14 ± 0.46 (1-BuOH)	17.50 ± 0.43	10.68 ± 0.91	55.7	1.04	0.29 ± 0.04	106.8
	1.00	0.89 ± 0.80	20.63 ± 0.24	9.96 ± 0.15 (1-BuOH)	13.99 ± 0.19	12.26 ± 0.44	49.8	0.81	0.23 ± 0.02	94.7
	2.12	2.00 ± 0.13	22.34 ± 0.37	10.71 ± 0.24 (1-BuOH)	14.91 ± 0.44	11.01 ± 0.09	53.4	0.97	0.20 ± 0.02	98.3
	5.26	1.85 ± 0.63	23.37 ± 1.67	10.71 ± 1.07 (1-BuOH)	17.55 ± 0.73	12.25 ± 0.69	53.4	0.87	0.25 ± 0.03	106.5
										
Glucose + 2-Me-1-butyrate	0.09	0.75 ± 0.08	24.43 ± 0.45	6.85 ± 0.32 (2-Me-1-BuOH)	18.02 ± 0.83	13.39 ± 1.54	34.3	0.51	0.25 ± 0.01	104.4
	0.34	0.76 ± 0.24	27.00 ± 1.85	8.15 ± 0.29 (2-Me-1-BuOH)	15.11 ± 0.43	13.36 ± 0.32	40.8	0.61	0.20 ± 0.01	106.0
	1.00	1.35 ± 0.06	24.19 ± 0.62	7.22 ± 0.24 (2-Me-1-BuOH)	11.91 ± 0.30	14.77 ± 0.51	36.1	0.49	0.18 ± 0.00	98.6
	2.12	2.04 ± 0.05	24.70 ± 0.72	7.24 ± 0.29 (2-Me-1-BuOH)	12.03 ± 0.77	15.09 ± 0.23	36.2	0.48	0.22 ± 0.02	98.4
	5.26	1.69 ± 0.49	24.63 ± 1.17	7.28 ± 0.69 (2-Me-1-BuOH)	11.92 ± 2.66	16.13 ± 1.10	36.4	0.45	0.20 ± 0.01	99.9
										
Glucose + 3-Me-1-butyrate	0.09	0.64 ± 0.05	19.18 ± 1.70	4.07 ± 0.29 (3-Me-1-BuOH)	14.10 ± 1.29	16.42 ± 0.69	20.4	0.25	0.26 ± 0.09	89.6
	0.34	1.09 ± 0.05	29.08 ± 1.88	5.89 ± 0.37 (3-Me-1-BuOH)	12.55 ± 0.63	16.17 ± 0.48	29.5	0.36	0.22 ± 0.02	106.1
	1.00	1.41 ± 0.06	25.62 ± 0.70	5.15 ± 0.11 (3-Me-1-BuOH)	9.83 ± 0.13	17.76 ± 0.92	25.8	0.29	0.18 ± 0.02	97.3
	2.12	1.92±0.26	25.78±3.80	4.92 ± 0.54 (3-Me-1-BuOH)	9.14 ± 1.04	18.09 ± 1.08	24.9	0.27	0.19 ± 0.01	96.6
	5.26	2.62 ± 0.30	25.72 ± 1.37	5.62 ± 0.03 (3-Me-1-BuOH)	9.81 ± 0.43	16.93 ± 0.03	28.1	0.33	0.22 ± 0.00	96.8

ND – Not detected; NA – Not applicable.

**Table 5:** Impact of glucose concentration on end product formation after 5 days from cultures of *T. pseudethanolicus* in the presence of (A) 1-propionate (B) 1-butyrate (C) 2-methyl-1-propionate bioconversion. Additionally, the percent of glucose consumed is shown (%C). Standard deviation is presented as error bars.

		**Analyte** (mmol/L)							
Substrate + carboxylic acid (20 mM)	Glucose (mM)	Hydrogen	Ethanol	Alcohol	Acetate	Carboxylic acid	Carboxylic acid conversion (%)	Optical Density (600 nm)	Carbon balance (%)
Glucose	0	0.13 ± 0.04	1.08 ± 0.14	ND	3.34 ± 0.20	ND	NA	0.23 ± 0.01	NA
	10	1.07 ± 0.21	12.44 ± 0.27	ND	4.24 ± 0.26	ND	NA	0.30 ± 0.01	83.4
	20	2.26 ± 0.43	32.48 ± 1.38	ND	7.03 ± 1.13	ND	NA	0.28 ± 0.02	98.8
	30	4.02 ± 0.27	49.23 ± 1.72	ND	15.46 ± 0.63	ND	NA	0.39 ± 0.03	107.8
	40	5.26 ± 0.14	48.63 ± 0.64	ND	25.33 ± 0.78	ND	NA	1.13 ± 0.24	92.5
Glucose +1-propionate	0	1.16 ± 0.26	1.94 ± 0.19	3.41 ± 0.27 (1-PrOH)	4.07 ± 0.53	17.37 ± 0.85	17.1	0.37 ± 0.10	103.9
	10	1.43 ± 0.97	8.67 ± 0.10	9.15 ± 0.71 (1-PrOH)	12.58 ± 1.05	10.59 ± 1.31	45.8	0.45 ± 0.12	102.5
	20	1.95 ± 0.57	17.88 ± 1.47	12.36 ± 1.34 (1-PrOH)	16.23 ± 1.12	7.86 ± 0.75	61.8	0.34 ± 0.01	90.6
	30	1.80 ± 0.42	35.14 ± 1.37	13.56 ± 0.83 (1-PrOH)	17.49 ± 0.57	6.57 ± 1.59	67.8	0.46 ± 0.05	91.0
	40	1.15 ± 0.52	30.87 ± 1.51	14.81 ± 1.57 (1-PrOH)	19.96 ± 2.95	5.25 ± 0.34	74.1	1.14 ± 0.17	88.6
Glucose +2-methyl-1-propionate	0	1.03 ± 0.14	2.59 ± 0.25	1.29 ± 0.18 (2-Me-1-PrOH)	3.81 ± 0.72	19.16 ± 1.80	6.4	0.31 ± 0.05	102.3
	10	1.64 ± 0.37	11.29 ± 1.83	5.90±0.40 (2-Me-1-PrOH)	8.66 ± 0.56	14.70 ± 1.70	29.5	0.51 ± 0.08	101.4
	20	1.89 ± 0.13	22.52 ± 3.41	8.79±0.09 (2-Me-1-PrOH)	11.65±0.14	10.35±1.09	44.0	0.45±0.08	88.9
	30	1.86 ± 1.13	32.71 ± 2.78	9.90 ± 0.40 (2-Me-1-PrOH)	13.48 ± 0.22	9.14 ± 0.86	49.5	0.71 ± 0.09	81.5
	40	1.70 ± 0.79	43.81 ± 3.57	10.67 ± 0.77 (2-Me-1-PrOH)	14.44 ± 1.07	6.54 ± 1.57	53.4	1.17 ± 0.16	94.3
Glucose +1-butyrate	0	1.14 ± 0.15	1.83 ± 0.07	1.18 ± 0.05 (1-BuOH)	3.64 ± 0.58	15.78 ± 2.17	5.9	0.31 ± 0.08	84.8
	10	1.95 ± 0.10	10.23 ± 3.36	4.26 ± 1.75 (1-BuOH)	7.25 ± 0.61	13.96 ± 1.32	21.3	0.30 ± 0.06	89.3
	20	1.36 ± 0.17	25.16 ± 1.13	8.07 ± 0.56 (1-BuOH)	9.86 ± 1.07	12.23 ± 1.64	40.4	0.47 ± 0.12	92.2
	30	1.12 ± 0.00	33.20 ± 0.78	9.14 ± 0.64 (1-BuOH)	10.27 ± 0.55	10.31 ± 0.42	45.7	0.60 ± 0.16	78.7
	40	1.61 ± 0.25	42.94 ± 4.56	10.14 ± 0.61 (1-BuOH)	12.73 ± 1.81	9.08 ± 0.33	50.7	0.90 ± 0.04	93.6

ND – Not detected; NA – Not applicable.

**Table 6:** Impact of alcohol addition on end product formation after 5 days from cultures of *T. pseudethanolicus* from glucose (20 mM) in the presence of (A) ethanol (B) 1-propanol (C) 2-propanol (D) 1-butanol (E) 2-methyl-1-propanol (F) 2-methyl-1-butanol (G) 1-pentanol (H) 1-hexanol.

		Analyte (mmol/L)			
Alcohol	% (v/v)	Hydrogen	Ethanol	Acetate	Optical Density (600 nm)
Ethanol	0	2.93 ± 0.07	26.28 ± 1.35	6.04 ± 0.10	0.38 ± 0.07
	0.5	10.55 ± 1.33	ND	10.80 ± 1.57	0.39 ± 0.07
	1	7.34 ± 1.70	ND	10.16 ± 0.45	0.20 ± 0.03
	2	10.00 ± 0.66	ND	12.26 ± 1.40	0.27 ± 0.00
	3	6.38 ± 0.21	ND	8.11 ± 0.10	0.19 ± 0.00
	4	3.86 ± 0.41	ND	5.32 ± 0.87	0.24 ± 0.08
	5	1.01 ± 0.15	ND	3.70 ± 0.29	0.31 ± 0.03
	7	0.48 ± 0.03	ND	1.63 ± 0.25	0.21 ± 0.07
					
1-Propanol	0	2.93 ± 0.07	26.28 ± 1.35	6.04 ± 0.10	0.38 ± 0.07
	0.5	7.82 ± 1.06	ND^a^	10.98 ± 0.41	0.57 ± 0.11
	1	7.38 ± 2.60	ND^a^	12.45 ± 0.47	0.31 ± 0.02
	2	5.36 ± 0.38	ND^a^	7.02 ± 0.53	0.28 ± 0.02
	3	0.00 ± 0.00	ND^a^	1.58 ± 0.03	0.23 ± 0.05
	4	0.00 ± 0.00	ND^a^	1.21 ± 0.20	0.26 ± 0.11
	5	0.00 ± 0.00	ND^a^	1.05 ± 0.05	0.25 ± 0.07
	7	0.00 ± 0.00	ND^a^	1.13 ± 0.18	0.38 ± 0.07
					
2-Propanol	0	2.93 ± 0.07	26.28 ± 1.35	6.04 ± 0.10	0.38 ± 0.07
	0.5	3.05 ± 0.14	ND^a^	4.09 ± 0.64	0.55 ± 0.09
	1	2.79 ± 0.23	ND^a^	2.96 ± 0.03	0.38 ± 0.07
	2	5.05 ± 0.17	ND^a^	4.28 ± 0.69	0.54 ± 0.11
	3	10.19 ± 0.02	ND^a^	3.96 ± 0.04	0.31 ± 0.16
	4	0.00 ± 0.00	ND^a^	1.30 ± 0.10	0.40 ± 0.17
	5	0.00 ± 0.00	ND^a^	1.39 ± 0.12	0.34 ± 0.05
	7	0.00 ± 0.00	ND^a^	1.06 ± 0.14	0.30 ± 0.02
					
1-Butanol	0	2.93 ± 0.07	26.28 ± 1.35	6.04 ± 0.10	0.38 ± 0.07
	0.5	5.44 ± 0.33	3.00 ± 0.39	9.84 ± 0.36	0.28 ± 0.01
	1	0.06 ± 0.01	2.33 ± 0.07	2.96 ± 0.20	0.11 ± 0.05
	2	0.00 ± 0.00	2.27 ± 0.03	2.64 ± 0.05	0.05 ± 0.01
	3	0.00 ± 0.00	2.32 ± 0.06	2.51 ± 0.24	0.06 ± 0.01
	4	0.00 ± 0.00	2.29 ± 0.00	2.64 ± 0.09	0.06 ± 0.01
	5	0.00 ± 0.00	2.75 ± 0.64	2.43 ± 0.14	0.06 ± 0.01
	7	0.00 ± 0.00	2.42 ± 0.02	2.48 ± 0.16	0.06 ± 0.01
					
2-Methyl-1-propanol	0	2.93 ± 0.07	26.28±1.35	6.04±0.10	0.38 ± 0.07
	0.5	2.90 ± 0.25	24.11 ± 0.56	3.60 ± 0.43	0.26 ± 0.07
	1	0.00 ± 0.00	2.87 ± 0.37	2.44 ± 0.31	0.22 ± 0.06
	2	0.00 ± 0.00	2.34 ± 0.07	2.63 ± 0.03	0.11 ± 0.05
	3	0.00 ± 0.00	1.88 ± 0.54	2.02 ± 0.68	0.09 ± 0.05
	4	0.00 ± 0.00	1.93 ± 0.54	1.36 ± 0.04	0.10 ± 0.04
	5	0.00 ± 0.00	1.73 ± 0.16	1.49 ± 0.20	0.05 ± 0.01
	7	0.00 ± 0.00	2.27 ± 0.12	1.44 ± 0.12	0.01 ± 0.00
					
1-Pentanol	0	2.93 ± 0.07	26.28 ± 1.35	6.04 ± 0.10	0.38 ± 0.07
	0.5	0.00 ± 0.00	2.33 ± 0.04	10.80 ± 1.57	0.17 ± 0.06
	1	0.00 ± 0.00	2.08 ± 0.15	10.16 ± 0.45	0.21 ± 0.01
	2	0.00 ± 0.00	1.51 ± 0.09	12.26 ± 1.40	0.19 ± 0.00
	3	0.00 ± 0.00	2.17 ± 0.10	8.11 ± 0.10	0.32 ± 0.07
	4	0.00 ± 0.00	2.24 ± 0.19	5.32 ± 0.87	0.32 ± 0.04
	5	0.00 ± 0.00	6.17 ± 0.01	3.70 ± 0.29	0.35 ± 0.11
	7	0.00 ± 0.00	6.99 ± 0.63	1.63 ± 0.25	0.53 ± 0.18
					
2-Methyl-1-butanol	0	2.93 ± 0.07	26.28 ± 1.35	6.04 ± 0.10	0.38 ± 0.07
	0.5	3.64 ± 0.84	25.02 ± 0.84	2.73 ± 0.04	0.39 ± 0.07
	1	0.00 ± 0.00	2.02 ± 0.03	2.82 ± 0.02	0.18 ± 0.03
	2	0.00 ± 0.00	1.91 ± 0.45	1.43 ± 0.34	0.16 ± 0.04
	3	0.00 ± 0.00	2.00 ± 0.10	1.57 ± 0.12	0.24 ± 0.00
	4	0.00 ± 0.00	2.19 ± 0.11	1.63 ± 0.03	0.33 ± 0.04
	5	0.00 ± 0.00	3.05 ± 0.07	1.41 ± 0.05	0.47 ± 0.02
	7	0.00 ± 0.00	2.57 ± 0.25	1.54 ± 0.17	0.40 ± 0.11
					
1-Hexanol	0	2.93 ± 0.07	26.28 ± 1.35	6.04 ± 0.10	0.38 ± 0.07
	0.5	0.00 ± 0.00	2.32 ± 0.09	2.76 ± 0.11	0.09 ± 0.02
	1	0.00 ± 0.00	2.31 ± 0.07	2.73 ± 0.12	0.07 ± 0.03
	2	0.00 ± 0.00	2.73 ± 0.07	2.80 ± 0.07	0.06 ± 0.03
	3	0.00 ± 0.00	2.51 ± 0.01	2.88 ± 0.08	0.04 ± 0.04
	4	0.00 ± 0.00	2.10 ± 0.04	2.73 ± 0.04	0.04 ± 0.01

a 1- and 2-propanol co-elute with ethanol.

**Table 7:** Enzyme activities using either NAD^+^ or NADP^+^ as a factor of culture of *T. pseudethanolicus* grown on glucose (20 mM) or glucose supplemented with carboxylic acid (20 mM) after 20 h at 65 °C. Values represent the average of triplicate determinations±standard deviation.

		Cofactor			
		NAD^+^		NADP^+^	
Growth conditions	Substrate	Specific activity (mU/ mg protein)	Relative activity (%)	Specific activity (mU/ mg protein)	Relative Activity (%)
Glucose	Control	1.5 ± 0.2	NA	1.3 ± 0.3	NA
	EtOH	25.4 ± 8.4	100.0^A^	36.8 ± 7.5	100.0^A^
	1-PrOH	25.9 ± 3.7	101.9^A^	41.7 ± 11.6	113.2^A^
	2-PrOH	23.2 ± 2.6	91.1^A^	31.4 ± 7.5	85.1^A^
	1-BuOH	25.9 ± 6.4	101.9^A^	53. 2 ± 11.1	144.6^A^
	2-BuOH	13.7 ± 3.5	54.0^A^	24.7 ± 9.2	67.0^A^
	2-Me-1-PrOH	26.2 ± 2.2	103.2^A^	40.2 ± 1.1	109.1^A^
	1-Pentanol	29.9 ± 7.9	117.8^A^	63.6 ± 6.6	172.6^A^
	2-Pentanol	18.1 ± 3.5	71.4^A^	32.2 ± 1.8	87.5^A^
	2-Me-1-BuOH	3.7 ± 13.7	14.6^A^	35.0 ± 5.1	95.0^A^
	3-Me-1-BuOH	2.5 ± 10.8	9.8^A^	34.1 ± 3.3	92.6^A^
	1-Hexanol	18.9 ± 11.4	74.4^A^	90.6 ± 13.5	246.0^A^
	2-Hexanol	18.9 ± 14.0	74.4^A^	27.4 ± 10.8	74.4^A^
	1-Heptanol	35.9 ± 2.6	141.4^A^	86.6 ± 7.9	235.3^A^
	1-Octanol	21.6 ± 1.8	85.2^A^	18.3 ± 2.1	49.7^A^
					
	Acetaldehyde	21.9 ± 12.4	100.0^B^	7.4 ± 1.6	100.0^B^
	Propionaldehyde	42.6 ± 8.3	194.5^B^	70.8 ± 13.0	956.8^B^
	Butyraldehyde	20.1 ± 11.0	91.8^B^	47.8 ± 0.0	645.9^B^
	2-Methyl-1-propionaldehyde	40.2 ± 29.8	183.5^B^	70.8 ± 10.8	956.8^B^
	Pentanaldehyde	79.4 ± 28.6	362.6^B^	73.0 ± 14.3	986.4^B^
	3-Methyl-butyraldehyde	13.1 ± 10.5	59.8^B^	73.9 ± 16.1	998.4^B^
	Hexaldehyde	69.9 ± 22.3	319.2^B^	80.0 ± 8.7	1080.1^B^
					
Glucose + 3-methyl-1-butyrate	Control	1.5 ± 0.2	NA	1.3 ± 0.3	NA
	EtOH	126.4 ± 7.2	100.0^A^	60.3 ± 6.6	100.0
	1-PrOH	105.8 ± 6.7	83.7^A^	73.6 ± 12.4	150.9
	2-PrOH	61.2 ± 3.0	48.4^A^	91.0 ± 3.9	141.1
	1-BuOH	87.1 ± 9.3	69.0^A^	85.0 ± 14.7	174.6
	2-BuOH	57.1 ± 6.8	45.2^A^	105.2 ± 7.7	146.1
	2-Me-1-PrOH	64.4 ± 0.5	50.9^A^	88.1 ± 5.3	186.0
	1-Pentanol	84.4 ± 8.1	66.8^A^	112.1 ± 29.5	166.4
	2-Pentanol	45.0 ± 7.6	35.6^A^	100.3 ± 23.9	138.4
	2-Me-1-BuOH	30.5 ± 3.7	24.1^A^	83.4 ± 10.7	90.7
	3-Me-1-BuOH	43.0 ± 7.0	34.0^A^	54.7 ± 7.3	130.8
	1-Hexanol	59.2 ± 10.9	46.9^A^	78.8 ± 17.3	126.5
	2-Hexanol	0.0 ± 0.0	0.0^A^	76.3 ± 2.8	212.7
	1-Heptanol	0.0 ± 0.0	0.0^A^	128.2 ± 10.2	110.8
	1-Octanol	0.0 ± 0.0	0.0^A^	66.8 ± 6.1	100.0
					
	Acetaldehyde	49.6 ± 2.2	100.0^B^	41.9 ± 1.5	100.0^B^
	Propionaldehyde	67.9 ± 6.0	136.9^B^	21.7 ± 4.5	51.8^B^
	Butyraldehyde	45.0 ± 7.9	90.7^B^	31.8 ± 19.6	75.9^B^
	2-Methyl-1-propionaldehyde	76.1 ± 9.1	153.4^B^	32.5 ± 10.3	77.6^B^
	Pentanaldehyde	81.9 ± 13.2	165.1^B^	22.1 ± 2.3	52.7^B^
	3-Methyl-butyraldehyde	65.4 ± 12.9	131.9^B^	41.7 ± 6.9	99.5^B^
	Hexaldehyde	37.5 ± 5.1	75.6^B^	79.5 ± 9.6	189.7^B^
					
Glucose + 1-pentanoic acid	Control	1.5 ± 0.2	NA	1.3 ± 0.3	NA
	EtOH	55.5 ± 8.8	100.0^A^	80.3 ± 14.3	100.0^A^
	1-PrOH	66.6 ± 1.6	120.0^A^	77.0 ± 6.0	96.0^A^
	2-PrOH	45.3 ± 11.5	81.6^A^	62.3 ± 6.8	77.6^A^
	1-BuOH	54.0 ± 4.9	97.4^A^	83.0 ± 28.3	103.4^A^
	2-BuOH	61.5 ± 3.3	111.0^A^	79.9 ± 6.2	99.5^A^
	2-Me-1-PrOH	55.3 ± 7.4	99.8^A^	77.7 ± 3.3	96.8^A^
	1-Pentanol	77.4 ± 7.3	139.6^A^	134.2 ± 44.2	167.1^A^
	2-Pentanol	58.6 ± 12.0	105.6^A^	79.8 ± 6.2	99.4^A^
	2-Me-1-BuOH	52.8 ± 24.6	95.1^A^	83.4 ± 7.9	103.9^A^
	3-Me-1-BuOH	47.1 ± 6.2	84.9^A^	65.2 ± 8.5	81.2^A^
	1-Hexanol	71.0 ± 2.0	128.0^A^	165.2 ± 9.1	205.8^A^
	2-Hexanol	62.1 ± 14.5	111.9^A^	86.2 ± 10.1	107.4^A^
	1-Heptanol	66.1 ± 23.2	119.1^A^	167.6 ± 14.8	208.8^A^
	1-Octanol	54.9 ± 4.3	99.1^A^	68.9 ± 10.9	85.8^A^
					
	Acetaldehyde	52.2 ± 12.0	100.0^B^	47.8 ± 3.4	100.0^B^
	Propionaldehyde	71.4 ± 5.6	136.8^B^	72.8 ± 8.5	152.3^B^
	Butyraldehyde	44.5 ± 13.2	85.2^B^	56.6 ± 2.1	118.4^B^
	2-Methyl-1-propionaldehyde	70.8 ± 22.2	135.6^B^	64.5 ± 16.6	134.9^B^
	Pentanaldehyde	92.6 ± 16.4	177.4^B^	71.7 ± 12.1	150.0^B^
	3-Methyl-butyraldehyde	49.5 ± 9.0	94.8^B^	64.6 ± 12.3	135.1^B^
	Hexaldehyde	74.3 ± 4.7	142.3^B^	60.4 ± 12.8	126.4^B^

A relative to ethanol.

B relative to acetaldehyde NA – not applicable.

## 3 Experimental Design, Materials and Methods

### 3.1 General methods

Yeast extract was obtained from Difco; nicotinamide cofactors were obtained from Megazyme while all other reagents were acquired from Sigma-Aldrich. Nitrogen gas was acquired from AGA and contained less than 5 ppm O_2_.

### 3.2 Microorganism and cultivation

*Thermoanaerobacter pseudethanolicus* (DSM 2355) was obtained from DSMZ culture collection. The strain was cultivated in serum bottles using the Basal Mineral (BM) medium prepared as previously described [2] using the Hungate technique [3,4]. The content and preparation of BM has been described earlier [2]. After media preparation it was transferred to serum bottles and autoclaved (121 °C) for 60 min. All heat sensitive components of the medium were added separately through filter (0.45 µm) sterilized solutions after autoclaving. Substrate concentration was 20 mM unless otherwise stated. All fermentations were done at 65 °C and at pH of 7.0 with a liquid–gas (L-G) ratio of 1:1 without agitation except stated otherwise. All growth experiments were performed using cultures taken from the exponential growth phase with inoculation volume of 2% (v/v). All cultivations were performed as triplicates and fermentation products were quantified after five days of cultivation unless stated otherwise.

### 3.3 Conversion of fatty acids in the presence of glucose

The strain was cultivated on glucose (20 mM) in the presence of added volatile fatty acids (20 mM). The strain was cultivated in Hungate tubes (18 × 150 mm) in BM containing glucose and the added acid. The acids added were formate, acetate, propionate, butyrate, 2-methyl-propionate, 2-methyl-1-butyrate, 3-methyl-1-butyrate, pentanoate, and hexanoate).

### 3.4 Kinetic experiments

Time course studies of glucose (20 mM) fermentation as the sole carbon source as well as with supplementation of exogenously added c 1-butyrate (20 mM) or 3-methyl-1-butyrate (20 M) were done in 125 mL serum bottles over a period of 120 h. Samples (1 mL) were taken periodically for the analysis of volatiles and gases (0.2 mL headspace gas). The strain was cultivated over a period of 7 days.

### 3.5 Effect of initial pH on glucose fermentation and carboxylic acid reduction

To study the influence of initial pH of the cultivation medium on the end product formation from glucose with exogenously added carboxylic acids (1-propionate, 1-butyrate, 2-methyl-1-butyrate, and 3-methyl-1-butyrate), the strain was grown in Hungate tubes (18 × 150 mm) in BM medium supplemented with glucose (20 mM) and 20 mM the acids (from stock solutions titrated to pH 7.0 ± 0.3) at pH ranging from pH 5.0 to 8.5 (in 0.5 pH unit increments). The pH of the cultivation broth was titrated to the desired pH using either 6 M NaOH and HCl prior to sterilization.

### 3.6 Effect of liquid–gas phase ratio on end product formation

*T. pseudethanolicus* was cultured in 125 mL serum bottles (118.5 mL nominal volume with butyl rubber septa inserted) with a defined L-G phase ratio; bottles were which were filled with a specific final volume of media to give defined L-G values of 0.09, 0.34, 0.98, 2.12, or 5.62. All cultivations otherwise contained glucose (20 mM) and one of the following fatty acids: 1-propionate, 1-butyrate, 2-methyl-1-butyrate, and 3-methyl-1-butyrate, (20 mM each).

### 3.7 Effects on inhibitors on end product formation

Effects of different concentrations of alcohols (ethanol, 1-propanol, 2-propanol, 1-butanol, 2-methyl-1-propanol, 2-methyl-1-butanol, 1-pentanol, and 1-hexanol) were tested using glucose as substrate (20 mM) in Hungate tubes (18 × 150 mm). The concentrations of alcohols used were 0.0, 0.5, 1.0, 2.0, 3.0, 4.0, 5.0, and 7.0% (*v/v*).

### 3.8 Effect of different initial glucose concentrations on end product formation

The strain was cultivated in Hungate tubes (18 × 150 mm) using four different concentrations (10, 20, 30 and 40 mM) of glucose with the addition of three different fatty acids (1-propionate, 1-butyrate, or 2-methyl-1-propionate).

### 3.9 ^13^C-labled experiment

BM medium supplemented with ^13^C1 butyrate and 20 mM glucose was syringe filtered into a 125 mL serum bottle with a liquid-gas phase ratio of 1:1. During cultivation, 1 mL samples were collected and frozen at −80 °C prior to analysis.

### 3.10 Analytical methods

Hydrogen, carboxylic acids, and low molecular weight alcohols were measured by gas chromatography as described earlier [2]. Glucose was analyzed by the 3,5-dinitrosalysylic acid method [5] in microplates. Optical density (OD) was quantified at a wavelength of 600 nm using a Shimadzu UV-1800 UV–visible spectrophotometer with quartz cuvettes (*l* = 1 m) against a water blank. Carbon-13 nuclear magnetic resonance (NMR) spectra were attained with a Bruker AV400 NMR Spectrometer; 1 mL of cell-free culture broth and 300 µL of D_2_O was added to achieve signal lock.

### 3.11 Enzyme assays

Cells were cultivated 1 L serum bottles fitted with butyl rubber septa containing 500 mL of BM media containing glucose (20 mM) and supplemented to afford 20 mM of carboxylic acid. Cells were harvested by centrifugation (4700 rpm, <4 °C) and rinsed 3X with rigorously degassed Tris–HCl buffer (50 mM, pH 7.5). The resultant cell pellets were resuspended in 10 mL of Tris–HCl to which one volume of glass beats (150–212 µm) were added; cells were lysed by vortexing three times (30 s followed by cooling on an ice bath for at least 2 min) and the cell debris removed by centrifugation as above. Lysed cell material was transferred to a sterile nitrogen flushed serum bottle.

Oxidative assays using NAD^+^ or NADP^+^ as a cofactor linked to nitroblue tetrazolium (NBT) reduction were performed using the method described by [6] with the modifications of [7]; briefly 50 µL of enzyme solution, 135 µL of reagent solution (containing 300 µM NAD^+^ or NADP^+^ and 0.13% w/v gelatin dissolved in 50 mM Tris–HCl, pH 8.0) supplemented to afford 5.5 mM of the relevant substrate, and PMS solution (80 µM) were added to microplates. Samples were incubated at 65 °C and the absorbance read every 5 minat a wavelength of 580 nm. A standard curve was constructed using NADH and the activity calculated according to the equation below where *v* is the sample volume in mL and *t* is time in minutes:

$$ADH\;activity\;\left(\text{mU}\;\text{per}\;\text{mL}\right)=\;\frac{nmol\;NADH}{v\cdot t}=nmol\;NADH\cdot \;2$$

## Limitations

Not applicable.

## Ethics Statement

The authors have read and follow the ethical requirements for publication in Data in Brief and confirming that the current work does not involve human subjects, animal experiments, or any data collected from social media platforms.

## CRediT authorship contribution statement

**Johann Orlygsson:** Supervision, Conceptualization, Methodology, Writing – review & editing, Writing – original draft. **Sean Michael Scully:** Methodology, Writing – review & editing, Investigation, Software.

## Data Availability

End product formation of glucose in the presence of organic acids by Thermoanaerobacter pseudethanolicus (Original data) (Mendeley Data)
